# Neuromuscular Profile of CrossFit^®^ Athletes: Part 1—Isometric and Ballistic Performance

**DOI:** 10.3390/jfmk11010118

**Published:** 2026-03-15

**Authors:** Diego A. Alonso-Aubin, Ester Jiménez-Ormeño, César Gallo-Salazar, Verónica Giráldez-Costas, Diana Ruiz-Vicente, Sara Zafra-Díaz, Francisco Areces-Corcuera, Carlos Ruiz-Moreno

**Affiliations:** 1 Strength Training and Neuromuscular Performance Research Group (STreNgthP), C/Castillo de Alarcón, 49, Villanueva de la Cañada, 28692 Madrid, Spain or ester.jimenez@uam.es (E.J.-O.); cgallo@ucjc.edu (C.G.-S.); vgiraldez@ucjc.edu (V.G.-C.); sara.zafrad@ucjc.edu (S.Z.-D.); fareces@ucjc.edu (F.A.-C.); 2 Faculty of Health Sciences—HM Hospitals, University Camilo José Cela, C/Castillo de Alarcón, 49, Villanueva de la Cañada, 28692 Madrid, Spain; diruiz@ucjc.edu (D.R.-V.); cruizm@ucjc.edu (C.R.-M.); 3 HM Hospitals Health Research Institute, 28015 Madrid, Spain; 4 Department of Physical Education, Sport and Human Movement, Universidad Autónoma de Madrid, 28049 Madrid, Spain; 5 Exercise Physiology Laboratory (GIDECS), Faculty of Health Sciences—HM Hospitals, University Camilo José Cela, C/Castillo de Alarcón, 49, Villanueva de la Cañada, 28692 Madrid, Spain; 6 Department of Social Sciences Applied to Sport, Physical Activity and Leisure, Polytechnic University of Madrid, C/Martín Fierro, 7, 28040 Madrid, Spain

**Keywords:** force plates, countermovement jump, maximal force, strength, athletic profiling, athletes, training, neuromuscular assessment

## Abstract

**Background**: CrossFit^®^ has gained widespread popularity as a high-intensity training modality, yet evidence describing neuromuscular performance characteristics in this population remains limited. This study aimed to evaluate isometric and ballistic strength profiles in trained CrossFit^®^ athletes and to identify sex-based differences in absolute and relative neuromuscular performance. **Methods**: Seventy-two athletes participated (41 males and 31 females) participated in the study, completing two maximal isometric mid-thigh pull (IMTP) tests and three countermovement jump (CMJ) tests within a single testing session. Assessments were conducted using a dual force plate system (Hawkin Dynamics, Westbrook, ME, USA). **Results**: In the IMTP, males exhibited substantially higher absolute isometric force outputs, including peak force (3059 ± 576 vs. 1899 ± 324 N; *p* < 0.001) and relative peak force (36.34 ± 6.74 vs. 30.99 ± 4.41 N/kg; *p* < 0.001). Rates of force development were also greater in males for both early (0–50 ms: 7665 ± 5420 vs. 4001 ± 3021 N/s; *p* < 0.001) and late phases (0–250 ms: 5350 ± 1832 vs. 3035 ± 886 N/s; *p* < 0.001). However, no significant sex differences were detected in time to peak force (2.31 ± 1.27 vs. 1.94 ± 1.04 s) or dynamic strength index (0.72 ± 0.12 vs. 0.73 ± 0.12 a.u.). In ballistic performance using CMJ, males achieved higher jump height (0.33 ± 0.07 vs. 0.23 ± 0.05 m; *p* < 0.001), jump momentum (215 ± 27.9 vs. 131 ± 19.1 kg·m/s; *p* < 0.001), and modified reactive strength index (0.46 ± 0.11 vs. 0.32 ± 0.08 a.u.; *p* < 0.001). Relative propulsive and braking forces were also moderately greater in males. Notably, sex differences were reduced when variables were normalized to body mass or peak force, indicating comparable relative neuromuscular function across sexes. **Conclusions**: These findings provide descriptive neuromuscular performance data for CrossFit^®^ athletes and show that sex-based differences primarily reflect disparities in absolute force-production capacity rather than intrinsic neuromuscular efficiency. Such insights may support more precise, evidence-informed, and sex-specific training prescriptions to optimize performance.

## 1. Introduction

CrossFit^®^ is a high-intensity training method that combines strength exercises, Olympic weightlifting movements, ballistic and plyometric activities, gymnastics, and metabolic conditioning to develop multiple physical capacities such as strength, endurance, and neuromuscular performance [1,2,3].

Muscular strength is a key component of performance in various sports [4], and in the specific case of CrossFit^®^, it is essential for both achieving competitive performance and preventing injuries [5]. Therefore, assessing strength in its different manifestations is crucial for understanding neuromuscular performance, which encompasses the efficiency and coordination of the nervous and muscular systems during movement, and for prescribing training effectively [6].

Maximal muscular strength can be assessed through various methods, including maximal isometric strength and maximal dynamic strength [6,7]. Maximal isometric strength, particularly measured using techniques like the isometric mid-thigh pull (IMTP) or isometric squat (ISQ), are highly valuable for evaluating an athlete’s strength and generating neuromuscular profiles across different athlete groups [8]. Moreover, the peak force (PF) in isometric tests, such as IMTP, demonstrates a strong correlation with other strength indicators like the one-repetition maximum (1-RM) in various exercises [9,10].

Importantly, research has established a significant relationship between IMTP PF and performance in weightlifting exercises such as the clean and jerk or snatch [11,12], which are integral to CrossFit^®^ workouts. Therefore, analyzing neuromuscular performance through IMTP can provide crucial insights into enhancing performance in these demanding exercises.

On the other hand, the assessment of ballistic strength has been widely employed in the scientific literature through the countermovement jump (CMJ), given its utility and strong correlation with other forms of strength [13]. The CMJ utilizes the stretch-shortening cycle (SSC), which is present in a wide range of sports activities, including running, jumping, and change in direction. In this sense, some WODs performed during the CrossFit^®^ Open Workouts from 2011 to 2022 [14], included exercises such as box jumps, burpees, or weightlifting movements like the snatch and clean and jerk, all of which require high levels of muscular power [15]. In this context, evaluating ballistic strength is particularly relevant for CrossFit^®^ athletes, as their WODs frequently include runs, changes in direction and plyometric exercises [16].

The dynamic strength index (DSI), calculated as the ratio of PF during the CMJ and the PF during IMTP, serves as a predictive measure of dynamic performance qualities, and it has proven to be a reliable and feasible tool for assessing neuromuscular performance [17].

Assessing and understanding performance across various domains of CrossFit^®^ athletes is crucial for optimizing training prescriptions and enabling meaningful performance comparisons among athletes. Consequently, some studies have published descriptive data on key aspects of neuromuscular performance, including maximal isometric strength, ballistic strength, and maximal dynamic strength, due to their significant impact on CrossFit^®^ performance [7,18,19].

Despite the growing popularity of CrossFit^®^, there remains a significant gap in research specifically addressing neuromuscular performance within this athletic population. Existing studies often lack a comprehensive evaluation of neuromuscular performance that incorporates various strength manifestations and provides descriptive data for both males and females.

The objective of the study was to evaluate the neuromuscular performance characteristics of CrossFit^®^ athletes, considering sex as a biological factor, to provide descriptive data on isometric and ballistic strength expressions and support the development of more effective and safer training prescriptions. These metrics would provide practical reference values to individualize training, monitor progress, optimize performance, and reduce injury risk, thereby supporting more effective and personalized CrossFit^®^ training strategies.

Additionally, the study aimed to determine whether differences exist in absolute and relative neuromuscular performance between males and females.

It was hypothesized that male CrossFit^®^ athletes would exhibit greater absolute neuromuscular performance than female athletes, while no significant sex-related differences would be observed in relative neuromuscular performance when values are normalized to body mass.

## 2. Materials and Methods

### 2.1. Study Design

An observational cross-sectional study was conducted in a single session where athletes performed a maximal isometric test using the mid-thigh pull (IMTP) and a ballistic test using the countermovement jump (CMJ). All tests were conducted by the athletes in three different CrossFit^®^ boxes in July 2025, ensuring equity in athletes’ fitness levels.

### 2.2. Participants

Seventy-two CrossFit^®^ athletes participated in the study; 41 males (age: 35.78 ± 8.23 years; height: 1.77 ± 0.06 m; body mass: 84.79 ± 11.08 kg; CrossFit^®^ experience: 4.32 ± 1.24 years) and 31 females (age: 36.61 ± 8.41 years; height: 1.62 ± 0.06 m; body mass: 61.02 ± 7.58 kg; CrossFit^®^ experience: 3.00 ± 2.18 years). All participants were actively engaged in competitive CrossFit^®^ and trained with a performance-oriented objective. Based on the Participant Classification Framework proposed by McKay et al. [20], and considering available training and competition information, participants would be most appropriately classified as Tier 2: Trained/Developmental. Exclusion criteria included a musculoskeletal injury in the previous 12 months or any medical condition that could limit exercise performance. Before starting, all participants provided an informed consent, being fully briefed on the purpose of the study, its risks, and benefits, and were made aware that they could withdraw at any time. The study was conducted in accordance with the ethical principles outlined in the Declaration of Helsinki (2013), and it was approved by the ethics committee of University Camilo José Cela (ID: 11_24_CROSSFITRM).

### 2.3. Procedures

The athletes attended their CrossFit^®^ box to complete all assessments in a single session under the same environmental conditions (20–25 °C and 40–50% humidity) and they were instructed to not alter their habitual dietary supplementation routines prior to testing. Prior to the evaluations, athletes completed a standardized warm-up protocol, followed by measurements of isometric maximal strength (IMTP) and ballistic strength (CMJ). For the IMTP and CMJ measurements, a fifth-generation dual force plate system (Hawkin Dynamics, Westbrook, ME, USA) was used with a sampling frequency of 1000 Hz. Jump height was calculated using the impulse–momentum method derived from the vertical ground reaction force–time data recorded by the force plates. Net vertical impulse was obtained by integrating the force signal after subtracting body weight, and take-off velocity was subsequently calculated as impulse divided by body mass. Jump height was then determined from take-off velocity using the kinematic equation (h = v^2^/2 g). This approach has been shown to provide a valid and reliable estimate of vertical jump performance and minimizes potential errors associated with flight-time-based calculations [21].

All tests were supervised by a Certified Strength and Conditioning Specialist (CSCS) who has 15 years’ experience in testing and training in strength athletes and data were collected using the Hawkin Dynamics software (APP: v10.0.1; Maths: v17.5.0), which connects the data from the force plate via Bluetooth to an Android tablet. The data were then transferred to the cloud for downloading in Microsoft Excel^®^ format for further analysis.

The standardized warm-up protocol included dynamic movements for both the upper and lower body, exercises using an unloaded barbell (e.g., squats, jumps, lunges, pulls), and a series of submaximal attempts for the specific tests (IMTP and CMJ).

#### 2.3.1. Maximal Isometric Strength Assessment

Participants followed a specific warm-up protocol based on the recommendations from Comfort et al. [22]. The warm-up for the IMTP test consisted of 3 attempts at 50%, 75%, and 90% of their perceived maximum effort to familiarize the participants with the protocol.

Before the assessment, each participant was instructed to stand on the platform with their feet centered on the bar at a width corresponding to their hip distance. This allowed for the estimation of the bar height in the isometric rack (Absolute Performance INC., Cardiff, Wales). The bar was positioned at a height corresponding to the start of the second pull or power position, with knee and hip angles ranging between 125 and 145° and 140–150°, respectively. Participants were also instructed to keep their torso upright, and all used a clean grip along with lifting straps to hold onto the immovable bar. They were instructed not to generate tension before the pull and to pull as hard and fast as possible, pushing vertically through the platform with both legs, while maintaining a straight torso to ensure maximal force application. For the evaluation, subjects were required to perform two maximum pulls lasting 5 s each, with a 1 min rest interval between attempts. The trial with the highest PF was used for statistical analysis.

Rate of force development (RFD) was included as a primary variable due to its ability to capture rapid force production capabilities, which are critical for explosive performance, and because its reliability has been shown to improve when assessed over longer time intervals (e.g., ≥200–250 ms), providing a balance between physiological relevance and measurement stability [23,24].

#### 2.3.2. Ballistic Strength Assessment

The participants performed a specific warm-up consisting of 3 CMJs at 50%, 75%, and 100% of their perceived maximum effort to familiarize themselves with the protocol.

Before the evaluation, each participant was instructed on the protocol [21], which involved stepping onto the force plate system and standing still in a bipedal position, with one foot on each platform for a few seconds, while keeping their hands on their hips in an akimbo position. Then, upon the evaluator’s signal, they were instructed to perform a jump with the highest possible height and speed (“jump as height as fast as possible”), with the option to flex their knees as needed.

For the evaluation, participants completed a total of three attempts with a 30 s rest between each attempt. The trial with the highest jump height was used for statistical analysis.

The inclusion of jump height, take-off velocity, peak relative braking force, peak relative propulsive force, jump momentum, and modified reactive strength index (mRSI) enables a comprehensive assessment of countermovement jump performance. Jump height and take-off velocity reflect overall performance outcomes, while jump momentum accounts for the influence of body mass, providing a more ecologically valid indicator of movement capability. Peak relative braking and propulsive forces offer insight into eccentric and concentric force-generation strategies, respectively, and mRSI integrates temporal and mechanical characteristics as an index of explosive efficiency. Together, these variables allow for a detailed characterization of the force–time profile [25,26].

#### 2.3.3. The Dynamic Strength Index (DSI)

DSI was calculated to quantify the ratio between dynamic and maximal isometric force production capability. For each participant, DSI was computed as the quotient of CMJ PF divided by IMTP PF (DSI = CMJ PF/IMTP PF) [27,28].

### 2.4. Statistical Analysis

Descriptive percentiles were derived from the sample distribution by aligning the cut-off points with the theoretical values of a standard normal distribution (z = −3, −2, −1, −0.5, 0, 0.5, 1, 2, and 3), which approximately correspond to the 0.15th, 3rd, 16th, 30.85th, 50th, 69.15th, 84th, 97th, and 99.85th percentiles, respectively. In addition, the first (25th) and third (75th) quartiles were included to facilitate the interpretation of inter-individual variability. This approach allows the dispersion of ballistic and isometric performance variables to be represented across the full range of standardized scores, providing a practical reference framework for classifying athletes’ relative performance levels.

Data are presented as mean ± standard deviation (SD), mean differences (95% confidence interval [95%CI]), percentiles and Hedges’ g effect size (ES). Statistical analyses were performed using Jamovi software (version 2.6.26). The Kolmogorov–Smirnov test was applied to verify the normality of the data, and all variables showed a normal distribution. Comparisons between sex (males vs. females) were performed for all variables related to isometric and ballistic performance. Student’s T-test was used to analyze variables with equal variances, whereas Welch’s T-test was used for those with unequal variances.

Hedges’ g was calculated to determine the magnitude of the effect between sexes, correcting for potential bias due to unequal group sizes. The thresholds used to interpret the effect sizes were defined as follows: ≤0.2, trivial; >0.2–0.5, small; >0.5–0.8, moderate; >0.8–1.2, large; and >1.2, very large. The level of statistical significance was set at *p* < 0.05. A post hoc power analysis was conducted using G*Power 3.1 for an independent samples *t*-test, with an effect size of g = 0.80, α = 0.05, and sample sizes of 41 and 31 participants per group. The achieved statistical power was 1−β = 0.91.

## 3. Results

Percentiles of both sexes for all variables regarding isometric and ballistic performance are shown in Table 1 and Table 2, respectively.

**Figure 1:**
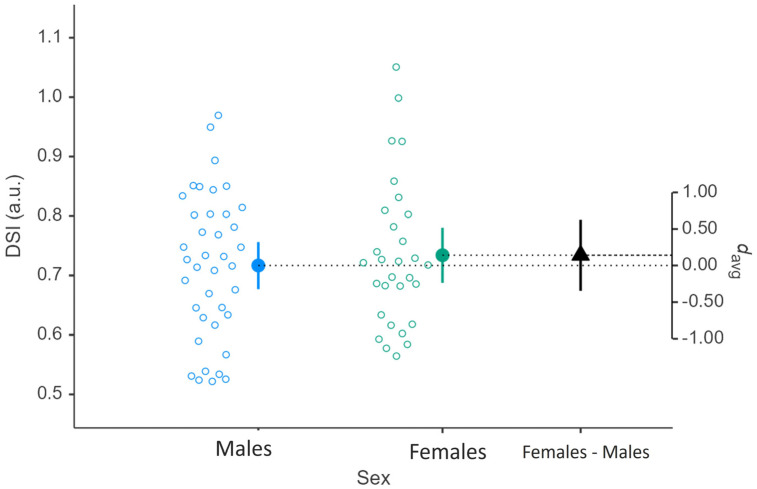
Interindividual responses for DSI of male and female CrossFit^®^ athletes derived from an Isometric Mid-Tigh Pull (IMTP) and Countermovement Jump (CMJ) test. Blue and green circles represent men and women individual values, respectively. Big dots depict means and vertical-colored lines represent standard deviations. Triangle represents standardized effect size (d_avg_ = Hedge’s g), and the vertical black line represents 95% IC.

Regarding the isometric performance, the comparative analysis between males and females revealed significant differences in most of the variables obtained from the IMTP test. Males exhibited higher values in PF (3059 ± 576 vs. 1899 ± 324 N; *p* < 0.001; Table 3) and relative PF (36.34 ± 6.74 vs. 30.99 ± 4.41 N/kg; *p* < 0.001; Table 3), showing very large and large effect sizes between sex, respectively. Similarly, males significantly outperformed females in RFD for both analyzed time intervals, RFD 0–50 ms (7665 ± 5420 vs. 4001 ± 3021 N/s; *p* < 0.001; Table 3) and RFD 0–250 ms (5350 ± 1832 vs. 3035 ± 886 N/s; *p* < 0.001; Table 3), with large and very large effect sizes, respectively. However, no sex-related differences were observed in either time to PF (2.31 ± 1.27 vs. 1.94 ± 1.04 s; *p* = 0.196; Table 3) or DSI (0.72 ± 0.12 vs. 0.73 ± 0.12 a.u.; *p* = 0.56; Figure 1).

As for ballistic performance, the comparative analysis between males and females revealed significant differences in all variables derived from the CMJ. Specifically, males showed substantially higher values in jump height (0.33 ± 0.07 vs. 0.23 ± 0.05 m; Table 4), jump momentum (215 ± 27.9 vs. 131 ± 19.1 kg·m/s; Table 4), and mRSI (0.46 ± 0.11 vs. 0.32 ± 0.08 a.u.; Table 4) compared to females (*p* < 0.001), with very large effect sizes. Significant differences were found in takeoff velocity (2.52 ± 0.27 vs. 2.12 ± 0.20 m/s; *p* < 0.001; Table 4), with a very large effect size. Moreover, variables related to relative propulsive PF (25.72 ± 4.64 vs. 22.65 ± 3.23 N/kg) and relative braking PF (24.04 ± 4.65 vs. 22.06 ± 3.18 N/kg) were also greater in males compared to females (*p* < 0.05; Table 4), showing small-to-moderate effect sizes.

## 4. Discussion

The objective of the study was to evaluate the neuromuscular performance characteristics of CrossFit^®^ athletes, considering sex as a biological factor, to provide descriptive data on isometric and ballistic strength expressions and support the development of more effective and safer training prescriptions. Additionally, the study aimed to determine whether differences exist in absolute and relative neuromuscular performance between males and females.

The present study fills an existing research gap by providing novel absolute and relative descriptive neuromuscular data for male and female CrossFit^®^ athletes, confirming significant sex-based differences in isometric and ballistic strength performance.

Regarding the comparison of isometric performance between males and females showed clear differences in IMTP metrics. As expected, males achieved higher PF and relative PF than females, which aligns with the typical differences in muscle mass and maximal strength observed between sexes [27,29]. These findings are consistent with the well-established influence of greater muscle mass, cross-sectional area, and higher maximal voluntary activation typically observed in males, which collectively contribute to superior maximal force–producing capabilities [30].

The capacity to rapidly generate force is critical for ballistic tasks and explosive movements—such as Olympic lifts and plyometric exercises—which are common in CrossFit^®^ training and competition. In this sense, males also demonstrated significantly greater RFD across both early (0–50 ms) and late (0–250 ms) contraction phases. These differences can be explained primarily by the greater absolute maximal strength typically observed in males [31].

The relative rate of force development (RFD/PF) is a more specific indicator of neuromuscular function, as it minimizes the influence of maximal strength and better reflects the intrinsic speed of force production driven by neural and contractile factors [32,33]. In line with previous research, expressing RFD relative to maximal force or body mass in the present study reduced or eliminated sex-related differences, suggesting similar relative explosive neuromuscular capacity between males and females, as reported in other studies [34].

These sex-based differences in absolute isometric strength and RFD highlight the need for individualized training strategies in CrossFit^®^ athletes. Coaches can use these data to adjust loading and progression, emphasizing maximal and rapid force development in female athletes while managing higher absolute loads and fatigue in males, thereby improving training effectiveness and safety.

Regarding the of ballistic strength assessment, differences were observed between males and females in jump height, jump momentum, relative braking and propulsive peak forces, as well as in mRSI. These findings are consistent with previous studies showing that males typically achieve higher values, primarily due to a greater capacity to generate force resulting from higher muscle mass [27,35]. Additionally, biomechanical and technical factors—such as execution velocity and coordination, have also been reported as contributors to these differences [36]. In this regard, it can also be observed that jump momentum is higher in males, as they are able to achieve greater take-off velocities.

In the case of mRSI, we show that males display higher values than females, as jump height is greater in males. This result may be a consequence of the higher jump height observed in males rather than differences in take-off time, as well as factors such as maximal strength, myotendinous stiffness, and jump strategies [37,38]. In general, the results indicate that males exhibit greater ability to generate and apply force explosively as well as in the reutilization of the stretch–shortening cycle.

These findings have clear practical implications for ballistic training in CrossFit^®^ athletes. Coaches can use sex-specific descriptive values to better individualize plyometric and jump-based training, adjusting intensity, volume, and complexity according to the athlete’s force-production capacity. For female athletes, programs may place greater emphasis on developing maximal strength, jump technique, and stretch–shortening cycle efficiency to improve jump height and mRSI. For male athletes, training may focus on refining force application, coordination, and load management to maximize explosive performance while minimizing excessive mechanical stress. Overall, these data support more targeted and safer ballistic training prescriptions.

Interestingly, despite the pronounced differences in force-related variables, no significant sex-related differences were observed in time to PF or the DSI. The DSI is also a sport-specific variable, and other studies have reported significant sex differences within the same sports [39]. However, due to the neuromuscular demands inherent to each sport, the DSI may be similar among males and females within a given sporting discipline, which supports the findings of our study [40]. Moreover, the DSI has been shown to be a reliable and feasible metric in sport settings [17].

Likewise, the similar DSI values indicate comparable relationships between ballistic and isometric force production, implying that the capacity to translate maximal isometric strength into dynamic performance is proportionally equivalent between males and females. These findings highlight that while absolute neuromuscular outputs differ substantially, the relative neuromuscular profiles and strength qualities follow similar patterns across sexes.

Higher DSI values indicate a greater ability to utilize maximal strength in fast or explosive tasks, whereas lower values suggest a larger disparity between maximal force capacity and force expressed dynamically. This information can be used to guide individualized training strategies; for instance, athletes with low DSI may benefit from ballistic or plyometric training to enhance rapid force production, while those with high DSI may require maximal strength development to increase overall force capacity. Consequently, DSI provides practitioners with a practical and evidence-based metric to inform targeted performance interventions [41].

The main strength of this study is the comprehensive characterization of neuromuscular performance in trained CrossFit^®^ athletes through the combined assessment of isometric (IMTP) and ballistic strength variables (CMJ), providing novel and sport-specific descriptive data while considering sex as a biological factor. However, CrossFit^®^ performance is inherently multidimensional; therefore, IMTP and CMJ outcomes should not be interpreted as comprehensive indicators of competitive success, but rather as objective measures of one relevant component within the broader performance spectrum. Future research should integrate neuromuscular profiling with aerobics, endurance, and skill-based assessments to provide a more ecologically valid and holistic characterization of functional fitness athletes.

Nevertheless, some limitations should be acknowledged. The cross-sectional design limits causal inference regarding the mechanisms underlying the observed sex-related differences. Additionally, although all participants were actively training, neuromuscular assessments were conducted under real-world conditions and were not standardized to a specific competitive phase, meaning that measurements may reflect different points within individual training cycles rather than peak performance. Furthermore, participant classification was based on available demographic and training information, but objective competition- and performance-based indicators (e.g., competition ranking, qualification standards, or benchmark performance outcomes) were not collected. According to the Participant Classification Framework proposed by McKay et al. [20], the cohort would most appropriately correspond to Tier 2 (Trained/Developmental). Consequently, the findings should be interpreted within the context of trained CrossFit^®^ practitioners, and their generalizability to novice or higher-tier (elite/international-level) athletes may be limited.

Future studies should aim to longitudinally examine neuromuscular adaptations in CrossFit^®^ athletes across different training phases and competitive periods to better understand how isometric and ballistic strength characteristics fluctuate throughout the season and in response to targeted training interventions. Additionally, investigating the relationship between neuromuscular performance metrics and specific CrossFit^®^ performance outcomes (e.g., competition ranking, workout-specific results, or benchmark workouts) would help clarify the practical relevance of these variables for performance optimization. Further research could also explore how different training strategies or strength–power emphasis (e.g., maximal strength–oriented vs. velocity-based approaches) influence the dynamic strength index and rate of force development in this population. Finally, expanding descriptive datasets to include athletes of different competitive levels and age groups would enhance the generalizability and applied utility of neuromuscular benchmarks in CrossFit^®^ training and athlete monitoring.

## 5. Conclusions

This study provides descriptive data on the neuromuscular performance of trained CrossFit^®^ athletes, highlighting clear sex-based differences in isometric and ballistic strength. Males showed higher absolute force, greater rates of force development, and superior ballistic performance, largely explained by greater muscle mass and maximal strength. However, when normalized to peak force or body mass, most sex differences were reduced or disappeared, indicating comparable relative neuromuscular function. No sex differences were observed in dynamic strength index or time to peak force, suggesting similar proportional transfer of maximal strength to dynamic performance. Overall, these findings indicate that sex-related differences are driven mainly by absolute force capacity rather than neuromuscular efficiency, providing useful benchmarks to inform more precise and sex-specific training strategies in CrossFit^®^ athletes.

## Figures and Tables

**Table 1:** Percentile cut-off points from components of isometric performance in male and female CrossFit^®^ athletes derived from the isometric mid-thigh pull (IMTP) test.

Percentile	z	Peak Force IMTP (N)		Relative Peak Force IMTP (N/kg)		RFD 0–50 ms (N/s)		RFD 0–250 ms (N/s)		Time to Peak Force (s)		DSI (a.u.)	
		M	F	M	F	M	F	M	F	M	F	M	F
99.85th	+3	4618	2491	51.38	39.71	23,422	10,674	11,407	5212	5.42	4.29	0.97	1.05
97th	+2	4140	2353	50.68	38.27	21,724	10,368	7989	4445	4.88	4.18	0.94	1.01
84th	+1	3467	2205	43.11	35.9	12,449	7097	6992	3826	3.35	2.87	0.84	0.84
75th	+0.67	3356	2134	40.44	33.5	9935	5363	6468	3639	3.25	2.67	0.8	0.8
69.15th	+0.5	3313	2096	39.88	32.73	8539	5015	6108	3539	3.08	2.44	0.79	0.76
50th	0	3081	1960	35.37	30.87	6400	3245	5276	2884	2.15	1.84	0.73	0.72
30.85th	−0.5	2676	1750	33.09	29.28	4556	1996	3992	2633	1.80	1.22	0.65	0.68
25th	–0.67	2582	1735	31.75	27.83	3585	1398	3880	2576	1.29	1.11	0.63	0.65
3th	–2.00	2532	1617	26.49	24.17	1872	698	3006	1653	0.26	0.51	0.52	0.58
0.15th	−3.00	2094	1279	21.09	24.14	1624	557	2913	1076	0.18	0.41	0.52	0.56

IMTP, isometric mid-thigh pull test; mRSI, modified reactive strength index; DSI: Dynamic strength index; M: Males; F: Females.

**Table 2:** Percentile cut-off points from components of ballistic performance in male and female CrossFit^®^ athletes derived from a countermovement jump (CMJ).

Percentile	z	Jump Height (m)		Jump Momentum (kg·m/s)		Relative Peak Braking Force (N/kg)		Relative Peak Propulsive Force (N/kg)		mRSI (a.u.)	
		M	F	M	F	M	F	M	F	M	F
99.85th	+3	0.46	0.33	276	161	40.94	32.07	42.94	32.25	0.66	0.49
97th	+2	0.45	0.33	268	156	33.44	28.7	35.11	30.26	0.64	0.48
84th	+1	0.38	0.27	243	150	27.43	24.42	28.73	24.8	0.58	0.4
75th	+0.67	0.37	0.25	233	146	25.71	23.38	27.2	23.48	0.54	0.37
69.15th	+0.5	0.37	0.25	226	144	25.34	22.79	26.84	23.14	0.52	0.35
50th	0	0.34	0.23	216	138	23.54	21.8	25.6	22.18	0.47	0.29
30.85th	−0.5	0.29	0.20	197	121	21.93	20.06	23.96	21.03	0.42	0.28
25th	–0.67	0.27	0.2	196	114	21.69	19.68	22.75	20.86	0.4	0.27
3th	–2.00	0.22	0.17	168	101	16.87	18.29	19.48	18.83	0.25	0.2
0.15th	−3.00	0.21	0.17	165	98	15.86	17.96	17.07	18.63	0.24	0.19

mRSI, modified reactive strength index; M: Males; F: Females.

**Table 3:** Components of isometric performance in male and female CrossFit^®^ athletes derived from the isometric mid-thigh pull (IMTP) test.

	Males	Females	*p*-Value	ES	Mean Diff (IC95%)
Peak Force IMTP (N)	3059 ± 576	1899 ± 324	<0.001	2.45	1160 (941, 1378)
Relative Peak Force IMTP (N/kg)	36.34 ± 6.74	30.99 ± 4.41	<0.001	0.93	5.35 (2.61, 8.08)
RFD 0–50 ms (N/s)	7665 ± 5420	4001 ± 3021	<0.001	0.82	3663 (1608, 5719)
RFD 0–250 ms (N/s)	5350 ± 1832	3035 ± 886	<0.001	1.59	2315 (1645, 2986)
RFD 0–50 ms/Peak Force IMTP (1/s)	2.40 ± 1.46	2.19 ± 1.69	0.574	0.14	0.21 (−0.54, 0.97)
RFD 0–250 ms/Peak Force IMTP (1/s)	1.73 ± 0.40	1.62 ± 0.46	0.264	0.27	0.12 (−0.09, 0.32)
Time to Peak Force (s)	2.31 ± 1.27	1.94 ± 1.04	0.196	0.32	0.37 (−0.20, 0.94)

Diff., difference; ES, Hedges’g effect size.

**Table 4:** Components of ballistic performance in male and female CrossFit^®^ athletes derived from a countermovement jump (CMJ).

	Males	Females	*p*-Value	ES	Mean Diff (IC95%)
Jump Height (m)	0.33 ± 0.07	0.23 ± 0.05	<0.001	1.62	0.1 (0.07, 0.12)
Jump Momentum (kg·m/s)	215 ± 27.9	131 ± 19.1	<0.001	3.46	83.8 (72.1, 95.5)
Takeoff Velocity (m/s)	2.52 ± 0.27	2.12 ± 0.2	<0.001	1.63	0.40 (0.29–0.51)
Peak Relative Braking Force (N/kg)	24.04 ± 4.65	22.06 ± 3.18	0.048	0.49	1.98 (0.02, 3.95)
Peak Relative Propulsive Force (N/kg)	25.72 ± 4.64	22.65 ± 3.23	0.003	0.76	3.07 (1.10, 5.00)
mRSI (a.u.)	0.46 ± 0.11	0.32 ± 0.08	<0.001	1.50	0.14 (0.10, 0.19)

Diff., difference; ES, Hedges’g effect size.

## Data Availability

The original contributions presented in this study are included in the article. Further inquiries can be directed to the corresponding author.
